# Host genes and HIV: the role of the chemokine receptor gene CCR5 and its allele.

**DOI:** 10.3201/eid0303.970302

**Published:** 1997

**Authors:** J M McNicholl, D K Smith, S H Qari, T Hodge

**Affiliations:** Centers for Disease Control and Prevention, Atlanta, Georgia 30333, USA. jkm7@cdc.gov

## Abstract

Since the late 1970s, 8.4 million people worldwide, including 1.7 million children, have died of AIDS, and an estimated 22 million people are infected with human immunodeficiency virus (HIV)(1). During 1995 and 1996, major clinical and laboratory discoveries regarding HIV pathogenesis provided new hope for the prevention and treatment of HIV infection. One major discovery was that members of the chemokine receptor family serve as cofactors for HIV entry into cells. We describe the role of allelic polymorphism in the gene coding for the CCR5 chemokine receptor with regard to susceptibility to and disease course of HIV infection. We also examine the effect of this discovery on medical and public health practices.


### Host Genes and HIV: The Role of the Chemokine Receptor Gene CCR5 and Its Allele (Δ32 CCR5)

**Janet M. McNicholl, Dawn K. Smith, Shoukat H. Qari, and Thomas Hodge**
Centers for Disease Control and Prevention, Atlanta, Georgia, USA

Since the late 1970s, 8.4 million people worldwide, including 1.7 million children, have died of AIDS, and an estimated 22 million people are infected with human immunodeficiency virus (HIV)(1). During 1995 and 1996, major clinical and laboratory discoveries regarding HIV pathogenesis provided new hope for the prevention and treatment of HIV infection. One major discovery was that members of the chemokine receptor family serve as cofactors for HIV entry into cells. We describe the role of allelic polymorphism in the gene coding for the CCR5 chemokine receptor with regard to susceptibility to and disease course of HIV infection. We also examine the effect of this discovery on medical and public health practices.

### The HIV Epidemic: a Global Problem

The World Health Organization has estimated that 22.6 million people are infected with human immunodeficiency virus (HIV) as of mid-1996 (1). The highest prevalence of HIV occurs in parts of Asia and sub-Saharan Africa. In 1995, HIV-associated illnesses caused the deaths of 1.3 million people, including 300,000 children under 5 years of age. No protective vaccine or cure is available, and while prevention methods are reducing incidence in some countries, HIV disease is expected to increase. Earlier HIV infection diagnosis, inhibition of ongoing HIV replication with antiretroviral therapy (in industrialized countries), and prevention and treatment of opportunistic infections and cancers delay the onset of AIDS and increase the life expectancy of HIV-infected persons.

### An Overview of Host Genes and HIV Infection

Since the early years of the HIV epidemic, significant differences in the rate of disease progression have been observed in longitudinally followed HIV-infected persons. The role of genes of the human leukocyte antigen (HLA) system in determining the course of disease has been examined by using such measures as the CD4+ cell count or the length of time between HIV infection and AIDS (2-6). Several HLA genes or haplotypes appear to influence disease progression, although the effects are complex and may depend on interactions with other host genes.

Studies of the effect of host genes on susceptibility to HIV infection were facilitated by the identification of persons who were persistently exposed to HIV but remained uninfected (7-12). Before the discovery of the role of chemokine receptor gene polymorphism in HIV infection, only genes of the HLA system were thought to protect against HIV infection. For example, certain distributions of HLA class I alleles were observed in uninfected female commercial sex workers in Africa (13,14) and Thailand (11), who had been highly exposed to HIV; additional class I and II alleles that may be associated with remaining uninfected have been identified (6). Several mechanisms, some related to cytotoxic T-cell function, have been suggested to explain these findings (7,10,11,15).

Non-HLA genetic factors also influence susceptibility to HIV infection and the course of HIV disease. In 1996 and 1997, studies confirmed the protective role of homozygosity for a 32 base pair (bp) deletion in the chemokine receptor gene, CCR5 (Δ32 CCR5), against HIV infection (9,12,16-19). Until recent reports of HIV infection in three persons homozygous for the 32 bp CCR5

Address for correspondence: Janet McNicholl, Centers for Disease Control and Prevention, MS A-25, 1600 Clifton Rd. N.E., Atlanta, GA 30333 USA; fax: 404-639-2149; e-mail: jkm7@cdc.gov.

deletion (20-22), no cases of HIV infection had been reported in studies of more than 60 persons homozygous for the CCR5 32 bp deletion. Presence of one copy of the deleted CCR5 gene also influences the course of disease as the onset of AIDS occurs later for some heterozygous persons than for those homozygous for the wild type CCR5 (12,17-19,23). The discovery of the role of CCR5 alleles has prompted studies of the possible role of many other host genes in HIV infection (24-26).

### What Are Chemokine Receptors?

Chemokine receptors are cell surface proteins that bind small peptides called chemokines (27). Chemokines can be classified into three groups based on the number and location of conserved cysteines: C, CC, and CXC. Chemokine receptors are grouped into families on the basis of the chemokine ligands they bind: CC, CXC, or both (27; Table 1). Some receptors are promiscuous, while others are selective in terms of ligand binding. The receptors are widely distributed on hematopoietic and other cells, but the Duffy antigen of erythrocytes (DARC) is the only member expressed on cells of erythroid lineage. Several human chemokine receptors have been classified as such on the basis of similarity of gene sequences and predicted protein structures, but their ligands have not been identified (orphan receptors). Among these is the recently identified *TER1* gene (28).

The characteristic feature of all chemokine receptors is a serpentine 7 transmembrane-spanning domain structure, which is shared with other receptors; e.g., the rhodopsin and the thyrotrophin receptors (Figure 1). Extracellular portions are involved in chemokine binding, while intracellular portions are involved in cell signaling. The effect of receptor-ligand interactions is usually mediated through G-protein coupled interactions; results in alterations in cell

Table 1. Human chemokine receptors

| Receptors (Old names)[a] | Ligands | | Predominant expression/ Tissue distribution | Pathogens[b] | Chromosome location | GenBank Acc. # |
|---|---|---|---|---|---|---|
| | C-C chemokines | CXC chemokines | | | | |
| <u>CC Receptors</u> | | | | | | |
| CCR1 (CC CKR1) | MIP-1α, ß, RANTES, MCP-3 | | monocytes, T cells | | 3p21 | L10918 |
| CCR2A (MCP-1Ra) | MCP-1 | | T cells, basophils monocytes, | | 3p21 | U03882 |
| CCR2B (MCP-1Rb) | MCP-1, 3, 4 | | HIV-1 (NSI) | | | U03905 |
| CCR3 (CKR3) | Eotaxin, RANTES, MCP-2,3,4 | | eosinophil, basophils, microglial cells, and possibly monocytes; little expression in peripheral blood T-lymphocytes or dendritic cells | HIV-1 (NSI) | 3p21 | U28694 |
| CCR4 | TARC | | basophils, T cells | | 3p24 | X85740 |
| CCR5[c] (CC CKR5) | RANTES, MIP-1α, ß | | monocytes, dendritic cells, microglial cells, T cells | HIV-1, (NSI) HIV2 | 3p21 | U57840 |
| <u>CXC Receptors</u> | | | | | | |
| CXCR1 (IL-8 RA) | | IL-8 | neutrophils, NK cells | | 2q35 | M68932 |
| CXCR2 (IL-8 RB) | | IL-8, MGSA, *gro*-α, NAP-2, IP-10, ENA-78, Mig | | | | M73969 |
| CXCR3 | | IP-10, Mig | activated T cells | | | X95876 |
| CXCR4 (Fusin, LESTR, HUMSTR) | | SDF-1 | wide: $CD4^+$ and $CD4^-$ cells, monocytes, macrophages, dendritic cells, B cells; other tissues, e.g., brain, lung, spleen | HIV-1 (SI) HIV-2 | 2q21 | M99293 |

function such as activation, motion, or migration, usually along a chemokine concentration gradient; and varies depending on the chemokine bound and the cell type (27).

Some chemokine receptors have a role in infectious disease susceptibility or pathogenesis. Several of the CC or CXC receptors are used by HIV-1 or HIV-2 as entry cofactors (Table 1). DARC serves as a cofactor for entry of *Plasmodium vivax* into erythrocytes (29); and as is the case with the 32bp-deleted CCR5 and HIV, resistance to *P. vivax* malaria is associated with lack of DARC expression (29), probably due to polymorphism of the DARC gene promoter (30). Several viruses (Epstein-Barr virus, cytomegalovirus, and *Herpes virus samiri*) contain functional homologues of human chemokine receptors, which suggests that the viruses may use these receptors to subvert the effects of host chemokines (27). They may also serve as HIV entry cofactors for human cells, as observed for HCMV-US28 (31).

### Chemokine Receptors and HIV Infection

The first clue that chemokine-related events were important in HIV pathogenesis came from work in R. Gallo's laboratory, which showed that high levels of chemokines could inhibit HIV replication in vitro (32). Then a cohort of highly exposed, HIV-negative men had high circulating levels of several chemokines, such as RANTES, MIP-1α, and MIP-1ß (8). These data led to the hypothesis that chemokines might prevent HIV infection by binding to the elusive HIV entry cofactor. Since the discovery of CD4 in the 1980s as an HIV receptor, it had become apparent that other factors were required for HIV to enter cells. For example, mouse cells expressing CD4 could not be infected with HIV (33). Using a novel cloning strategy, E. Berger and colleagues first demonstrated in May 1996 that CXCR4, a chemokine receptor for which no ligand had yet been determined, was an entry cofactor for T-cell tropic or syncytium-inducing (SI) viruses (34).

Table 1. Human chemokine receptors (continued from p. 2)

| Receptors (Old names)[a] | Ligands: C-C chemokines | Ligands: CXC chemokines | Predominant expression/ Tissue distribution | Pathogens[b] | Chromosome location | GenBank Acc. # |
|---|---|---|---|---|---|---|
| <u>CC/CXC Receptor</u> | | | | | | |
| DARC (Duffy antigen) | RANTES, MCP-1, TARC etc. | IL-8, MGSA, *gro*-α *etc.* | endothelial cells, erythrocytes | *Plasmodium vivax* | 1 | U01839 |
| <u>Others</u> [d] | | | | | | |
| STRL33 | ND | ND | lymphoid tissues and activated T cells | HIV-1 | 3 | U73529 |
| HCMV-US28 | MIP-1α, ß, RANTES | | fibroblasts infected with CMV | HIV-1 | N/A | X17403 |
| ChemR1 | ND | ND | T lymphocytes, polymorphonuclear cells | | 3p21-24 | Y08456 |
| CMKBRL1 | ND | ND | neutrophils, monocytes, brain, liver, lung, skeletal muscles | | 3p21 | U28934 |
| *TER*1 | ND | ND | thymus, spleen | | 3p21 | U62556 |
| V28 | ND | ND | Neural and lymphoid tissue | | 3p21 | U20350 |
| D2S201E | ND | ND | wide, including cells of hemopoietic origin | | 2q21 | M99293 |
| BLR1 | ND | ND | B lymphocytes | | | X68149 |
| EBI1 | ND | ND | B lymphocytes | | | L08176 |
| GPR1,2,5 | ND | ND | | ND | | L36149 |

[a] New nomenclature for CC and CXC chemokine receptors was adopted at the Gordon Research Conference on Chemotactic Cytokines, June 23–28, 1996. [b]Pathogens using this receptor for infection. [c] The 32bp deleted allele of CCR5 has been referred to as CCR5-2 (19). [d]Chemokine receptor-like genes whose predicted proteins have 7 transmembrane domains.

Abbreviations: BLR1, Burkitt's lymphoma receptor-1; CMKBRL1, Chemokine ß receptor like-1; DARC, duffy antigen/receptor for chemokines; EBI1, Epstein-Barr virus-induced receptor; ENA78, epithelial-derived neutrophil-activating peptide-78; GPR, G protein coupled receptor; *gro*, growth related gene product; HCMV, human cytomegalovirus; HUMSTR, human serum transmembrane segment receptor; IL, interleukin; IP-10, interferon-gamma inducible 10kD protein; LESTR, leukocyte-expressed seven-transmembrane-domain receptor; MCP, monocyte chemotactic protein; Mig, monokine induced by interferon gamma; MIP, macrophage inflammatory protein; NSI, non-syncytium inducing; N/A, not applicable; NAP-2, neutrophil-activating protein-2; ND, not determined; RANTES, regulated on activation, normal T cell expressed and secreted; SDF-1, stromal cell-derived factor-1; STRL33, seven transmembrane-domain receptor from lymphocyte clone 33;TARC, thymus and activation regulated chemokine.

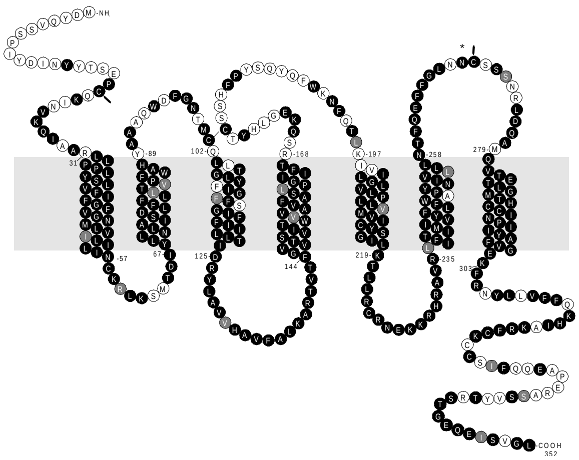

Figure 1. Predicted structure and amino acid sequence of CCR5. The typical serpentine structure is depicted with three extracellular (top) and three intracellular (bottom) loops and seven transmembrane (TM) domains. The shaded horizontal band represents the cell membrane. Amino acids are listed with a single letter code. Residues that are identical to those of CCR2b are indicated by dark shading, and highly conservative substitutions are indicated by light shading. Extracellular cysteine residues are indicated by bars, and the single N-linked glycosylation consensus site is indicated by an asterisk. Reprinted and modified with permission from the authors and Cell (39). Copyright (1996) Cell Press.

Shortly before this, Samson et al. had cloned CCR5 (then known as CC-CKR5) and shown that its ligands included RANTES, MIP-1α, and MIP-1ß (35). These data encouraged several groups to examine the role of CCR5 in HIV entry, and within weeks of the CXCR4 publication, five additional publications reported CCR5 and CCR3 as HIV entry cofactors for macrophage-tropic or non-syncytium-inducing (NSI) viruses (33,36-39) and CCR3 and CCR2b as entry factors for dual tropic HIVs (38,39). Recent studies of brain-derived microglial cells have shown that CCR5 and CCR3 permit entry of HIV into these cells (40).

The tropism of HIV strains appears to be determined in part by the way they use chemokine receptors (Table 1, Figure 2). In peripheral blood, although both T cells and monocytes express CXCR4 and CCR5 (41), HIV strains that use CXCR4 tend to infect predominantly T cells, while strains that use CCR5 infect T cells and monocytes. This tropism correlates with the ability of the virus to induce syncytia in T-cell lines. SI viruses (often present late in the course of HIV infection), tend to be CXCR4 tropic, while NSI viruses (which appear to be the viruses transmitted in vivo) tend to be CCR5 tropic (33,34,36-39). However, some primary SI viruses can use either CXCR4 or CCR5 (42), and some may use both coreceptors, as well as CCR2b or CCR3 (39). When classified based on chemokine receptor use, HIV types are independent of their genetic relatedness (43).

The exact mechanism by which HIV interacts with CXCR4 or CCR5 and by which this interaction, together with CD4 binding, leads to virus entry has not been clearly defined (44). It appears to involve the interactions of the V3 loop and other parts of the outer envelope protein gp120 (45-47) with extracellular domains of CCR5 or CCR2b (48,49) and may involve multistep

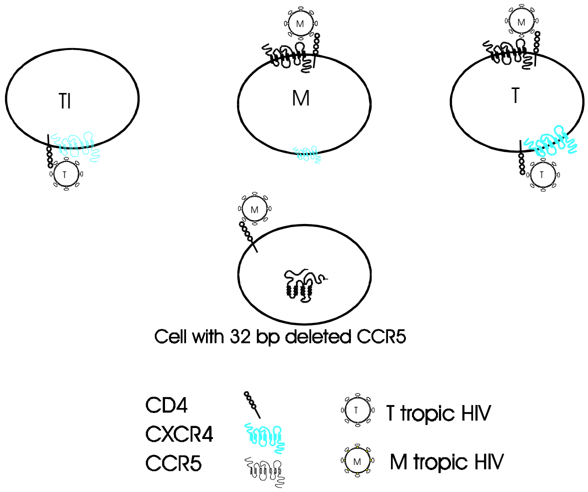

Figure 2. Chemokine receptors and cell tropism of HIV. Three cell types are illustrated, an in vitro passaged T-cell line (Tl), a monocyte/macrophage (M), and a circulating T-cell (T). T-cell lines express CXCR4 but not CCR5; macrophages and circulating peripheral blood T-cells express both receptors, although the amounts of CXCR4 are lower on macrophages (as indicated by the small CXCR4 symbol). M-tropic HIV, because of certain envelope amino acid sequences, binds to CCR5 and can enter both macrophages and circulating T cells. T-tropic HIV preferentially binds CXCR4 and enters T cells or T-cell lines. After binding to the chemokine receptor and to CD4, the viruses enter by fusion with the cell membrane. Cells with the 32bp deleted form of CCR5 do not express cell surface CCR5, and, although M-tropic HIV can bind CD4, it cannot enter the cell. If the cells express CXCR4, they can still be infected with T-tropic viruses (not shown). Note that this figure does not depict the actual size relationships of the proteins, cells, or viruses.

interactions with CD4, the chemokine receptor, and other cell surface components (50). Chemokines may inhibit HIV entry through receptor blockade, desensitization, sequestration, or internalization; through alterations in receptor affinity; or by inhibiting postbinding steps (51), such as phosphorylation, through G-coupled mechanisms.

### Chemokine Receptor Gene Polymorphism and Susceptibility to HIV Infection

In 1995 and 1996, researchers at the Aaron Diamond AIDS Research Center began studies of highly HIV-exposed seronegative men whose cells produced high levels of chemokines in vitro (8,52). Cells from several of these men were not infectable with primary or NSI type viruses, but were infectable with SI viruses (52). These data suggested some protective mechanism related to the CCR5 rather than to the CXCR4 receptor because of the chemokine ligand profile of the CCR5 receptor (Table 1) and stimulated studies that led to the cloning and restriction digestion of CCR5 in these persons (52). The novel deletion of 32bp in CCR5 reported by Liu et al. (9) in August 1996, to be present in three of the 15 HIV-exposed but seronegative men was also independently reported in September by Samson et al. (16) and Dean et al. (17; Table 2). In these studies, the distribution of homozygosity and heterozygosity of the deleted allele, in small and large cross-sectional or prospective studies of HIV-unexposed, -exposed, or -infected persons (mainly European or North American Caucasians) strongly supported the protective effect of the deletion. Since these initial observations, several studies of other populations have reported the same finding: an enrichment of the homozygous Δ32/Δ32 CCR5 genotype in highly HIV-exposed persons who remain uninfected (12,18,19; Table 2). In persons with well-documented HIV exposure, the prevalence of homozygosity for the Δ32/Δ32 CCR5 genotype increases with increasing HIV exposure (12), the wild type and Δ32 CCR5 alleles (referred to by some as CCR5-1 and CCR5-2 [19]) can be easily detected by polymerase chain reaction (PCR) techniques, with or without enzymatic digestion (9,12,16,17), or more recently, by heteroduplex studies (19). An example of the results of a PCR followed by restriction digestion is given in Figure 3. Persons who are homozygous for the wild type CCR5 (W) or the allele with the 32 bp deletion (Δ32) or heterozygous for both alleles can be easily distinguished because the deleted fragment reduces the size of the amplified product (Figure 3).

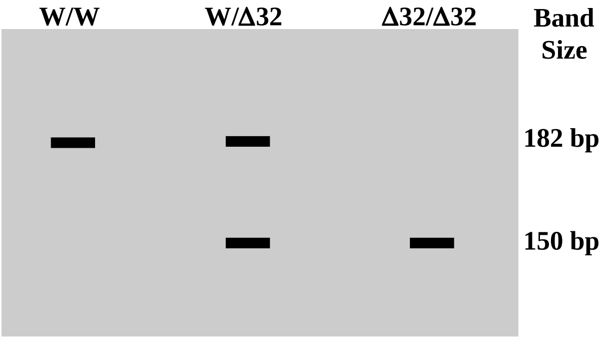

Figure 3. Differentiation of CCR5 genotypes by gel electrophoresis. Band patterns of persons with homozygous wild type (W/W), homozygous 32 bp deletion (Δ32/Δ32) or heterozygous W/Δ32 CCR5 genotypes are shown. PCR amplification of the C-terminal of the CCR5 gene, subsequent digestion with the EcoRI restriction enzyme, and agarose gel electrophoresis of the digested DNA yield a 182 bp band for the wild type CCR5 gene, a 150 bp band for the 32 allele, and both bands in the case of a heterozygous person.

The 32 bp deletion occurs at a site of a repeat motif in the CCR5 gene (16; Figure 4) and results in a frame shift in the coding sequence that produces a defective CCR5, which is not expressed on the surface of cells (Figures 2 and 5). The deletion results in the loss of three of the seven transmembrane domains, two of the three outer loops, and the intracellular signaling domain (Figure 5).

### Does the CCR5 32 Bp Deletion Provide Absolute Protection Against HIV Infection?

The genetic link to remaining HIV-negative in spite of continued HIV exposure was the subject of much discussion during the summer and fall of 1996. Was the protection absolute? Who should be tested for the gene? Would persons told they were homozygous for the deletion engage in high-risk behavior more frequently? Although at that point no one homozygous for the deletion had been found HIV-positive, the fact that CXCR4 gene and several other chemokine receptors could mediate HIV entry suggested caution in assuming that persons with the CCR5 Δ32/Δ32 genotype would be absolutely resistant to HIV infection. Since then, three HIV-infected persons with this genotype have been reported (20-22; Table 2); for one, the mode of

Table 2. Population studies of CCR5 genotypes

| Author | Population | Ethnicity | HIV Status | No. | [a]CCR5 Genotypes % (No.) W/W | W/ Δ32 | Δ32/Δ 32 | Δ32 allele freq. (%) |
|---|---|---|---|---|---|---|---|---|
| Liu et al. (9) | High-risk, USA | Caucasian | Neg | 15 | 80.0 (12) | 0 (0) | 20.0 (3) | 0.200 |
| | General, USA | Caucasian | Neg | 122 | 80.3 (98) | 19.7 (24) | 0 (0) | 0.098 |
| | General, S. Amer. | Venezuelan | Neg | 46 | 100 (46) | 0 (0) | 0 (0) | 0 |
| Samson et al. (16) | General, Europe | Caucasian | Neg | 704 | 82.7 (582) | 16.2 (114) | 1.1 (8) | 0.092 |
| | General, Asia | Japanese | Neg | 248 | 100 (248) | 0 (0) | 0 (0) | 0 |
| | General, Africa | Ctrl/W. African | Neg | 124 | 100 (124) | 0 (0) | 0 (0) | 0 |
| | AIDS clinics, Paris | Caucasian | Pos | 723 | 89.2 (645) | 10.8 (78) | 0 (0) | 0.054 |
| Dean et al.[b](17) | High-risk, USA | Mixed | Neg | 883 | 81.9 (724) | 15.6 (138) | 2.4 (21) | 0.10 |
| | High-risk, USA | Mixed | Pos | 1883 | 85.9 (1618) | 14.0 (264) | 0.0005 (1) | 0.071 |
| | High-risk, USA | African-Amer. | Mixed | 620 | 96.6 (599) | 3.4 (21) | 0 (0) | .017 |
| | High-risk, USA | Caucasian | Mixed | 1250 | | | | 0.115 |
| | Low-risk, USA | Caucasian | Mixed | 143 | | | | 0.080 |
| Huang et al.(12) | High-risk, USA | Caucasian | Pos | 461 | 79.8 (368) | 20.2 (93) | 0 (0) | 0.101 |
| | High-risk, USA | Caucasian | Neg | 446 | 78.0 (348) | 16.7 (82) | 3.6 | 0.128 |
| | Blood donors, USA | Caucasian, 95% | Neg | 637 | 85.2 | 13.3 | 1.4 | 0.08 |
| | General, Africa/Haiti | Black | Mixed | 137 | 100 | 0 (0) | 0 (0) | 0 |
| | General, Asia | China/Thai/ other | Mixed | 191 | 100 | 0 (0) | 0 (0) | 0 |
| Michael et al.(18) | High-risk, USA | Mixed | Pos | 406 | 87.5 (348) | 14.3 (58) | 0 (0) | 0.071 |
| | High-risk, USA | Mixed | Neg | 21 | 71.4 (15) | 9.5 (2) | 19.1 (4) | 0.238 |
| | Intermed.-risk, USA | Mixed | Neg | 240 | 78.3 (188) | 20.4 (49) | 1.3 (3) | 0.115 |
| Biti et al. (20) | High-risk, Australia | Caucasian | Pos | 265 | N/A[c] | N/A | 0.004 (1) | N/A |
| Theodoru et al. (22) | High-risk, Europe | | Pos | 412 | N/A | N/A | 0.002 (1) | N/A |
| Eugenolsen et al. (23) | High-risk, Denmark | Caucasian | Neg | 35 | 74 (26) | 20 (7) | 6 (2) | 0.157 |
| | High-risk, Denmark | Caucasian | Pos | 99 | 78 (77) | 22 (22) | 0 | 0.111 |
| | Blood donors, Denmark | Caucasian | Neg | 37 | 73 (27) | 24 (9) | 3 (1) | 0.149 |
| Zimmerman et al. (19) | Blood donors, N. America | Caucasian | Neg | 387 | 77.5 (300) | 21.7 (84) | 0.8 (3) | 0.116 |
| | | African-Am. | Neg | 294 | 94.2 (277) | 5.8 (17) | 0 (0) | 0.29 |
| | | Hispanic | Neg | 290 | 92.8 (269) | 6.9 (20) | 0.3 (1) | 0.38 |
| | | Asian | Neg | 164 | 99.4 (163) | 0.6 (1) | 0 (0) | 0.003 |
| | | Native Amer. | Neg | 87 | 83.9 (73) | 12.6 (11) | 3.4 (3) | 0.098 |
| | Blood donors, India | Tamil | Neg | 46 | 100 (46) | 0 (0) | 0 (0) | 0 |
| | Blood donors, W. Africa | Black | Neg | 40 | 100 (40) | 0 (0) | 0 (0) | 0 |
| | High risk, USA | Caucasian | Pos | 614 | 77.4 (475) | 22.6 (139) | 0 (0) | 0.113 |
| | High risk, USA | African-Amer. | Pos | 86 | 97.7 (84) | 2.3 (2) | 0 (0) | 0.012 |
| | High risk, USA | Hispanic | Pos | 45 | 93.3 (42) | 6.7 (3) | 0 (0) | 0.033 |
| | High risk, USA | Caucasian | Neg | 111 | 73.9 (82) | 21.6 (24) | 4.5 (5) | 0.153 |
| | High risk, USA | African-Amer. | Neg | 2 | 50.0 (1) | 0 (0) | 0.250 | |
| | High risk, USA | Hispanic | Neg | 12 | 91.7 (11) | 8.3 (1) | 0 (0) | 0.042 |

[a] CCR5 genotypes: W/W, homozygous wild type; W/ Δ32, heterozygous wild type/32bp deletion; Δ32/Δ 32, homozygous for the 32 bp deletion.

[b] Numbers updated to include additional Multicenter Hemophilia Cohort Study patients studied by O'Brien et al. (21) and Dean et al. (pers .comm.).

[c]Not available.

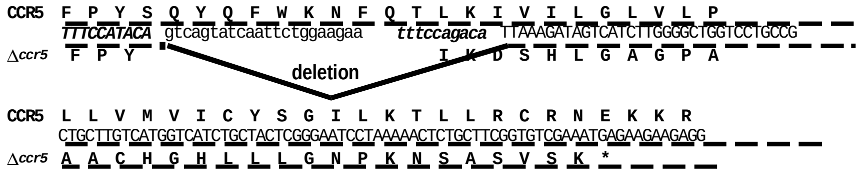

Figure 4. Partial CCR5 gene and amino acid sequence with 32 bp deletion. Nucleotide sequence of the CCR5 gene surrounding the deleted region, and translation into the normal receptor (top lines) or the truncated mutant (Δ 32 CCR5, bottom lines). The 10-bp direct repeat is represented in bold italics and the deleted nucleotides are represented in noncapitalized font.

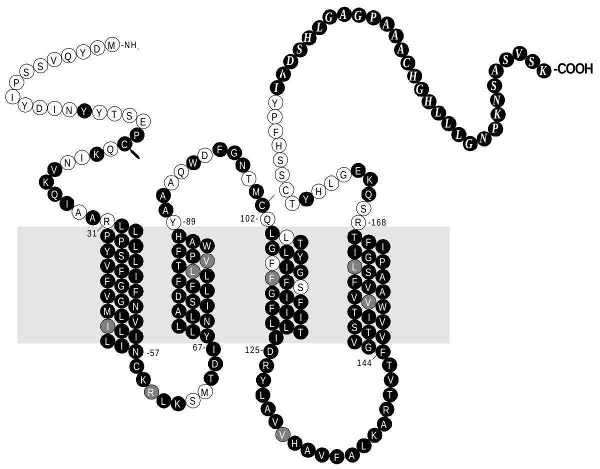

Figure 5. Predicted structure and amino acid sequence of the mutant form of human CCR5. The mutant protein lacks the last three transmembrane segments of CCR5, as well as the regions involved in G-protein coupling. The transmembrane organization is given by analogy with the predicted transmembrane structure of the wild-type CCR5, although the correct maturation of the mutant protein up to the plasma membrane has not been demonstrated. The shaded horizontal band represents membrane (s) of intracellular organelles. Amino acids represented in italic and dark shading correspond to unnatural residues resulting from the frame shift caused by the deletion. Figures 4 and 5 reprinted and modified with permission from the authors and Nature (16) Copyright (1996) Macmillan Magazines Ltd.

transmission was most likely blood products (21), while the only reported exposure risk for the other two was homosexual behavior (20,22). Sequences isolated from these persons suggested the SI virus phenotype, i.e., viruses that most likely used CXCR4 as entry cofactors (21; R. Ffrench, pers. comm.). As several other non-CCR5 receptors (CCR3 and CCR2b) are also HIV entry cofactors, even non-SI viruses might be transmissible in vivo in persons homozygous for the CCR5 32 bp deletion. Thus, HIV entry by non-CCR5 dependent mechanisms can occur in vivo, and persons who have the CCR5 Δ32/Δ32 genotype are not absolutely protected.

### Does CCR5 Gene Polymorphism Determine All Resistance to HIV?

The highest reported prevalence of homozygosity for the Δ32 CCR5 allele deletion in persons highly exposed to HIV but uninfected is 33% (9,12). However, most (96% according to one estimate [19]) highly exposed HIV-seronegative persons are not homozygous for the Δ32 CCR5 allele. Moreover, among exposed but persis-tently HIV-seronegative per-sons in Africa and Thailand, no one positive for the Δ32 CCR5 allele has been found (CDC unpub. data; S. Rowland-Jones, F. Plummer, pers. comm.). These data suggest that many mechanisms may contribute to remaining seronegative despite high HIV exposure: chemokine-related mechanisms, such as altered CCR5 levels on the cell surface (53) or the level of circulating chemokines; other immune system genes, such as those of the HLA system (10,11,14); and immune responses, such as those of cytotoxic T cells (7,15). The relative importance of these mechanisms in populations with differing modes of exposure or genetic backgrounds needs to be elucidated.

### CCR5 Polymorphism and Its Interaction with Other Factors in the Course of HIV Disease

Several large studies of the effect of CCR5 genotypes on the course of HIV disease have been published (Table 2). Dean et al. examined the course of HIV (time to AIDS) in several U. S. populations with different exposure to HIV (homosexuals, intravenous drug users, persons with hemophilia) and found that persons with one copy of the deleted CCR5 gene had a delayed (approximately 2 years longer) progression to AIDS when compared with those with the homozygous wild type genotype (17). Similar findings have been reported (19,23) in Caucasian HIV-positive homosexual men. Another study of HIV-positive homosexuals did not find such a striking effect of heterozygosity, although progression to AIDS was again slowed (12). One clue to the varying effects of the heterozygous CCR5 genotype on progression to AIDS in different groups comes from another U. S. study of male homosexuals, in which men with one copy of the Δ32 CCR5 genotype had delayed progression to AIDS, particularly if their circulating viruses were of the NSI phenotype (18). Paradoxically, once AIDS is diagnosed, persons with one copy of Δ32 CCR5 may have a more rapid disease course.[1] Thus, the effect of the CCR5 genotype on disease progression may depend on the phenotype and chemokine receptor use of circulating viruses. The finding that microglial cells can be infected with HIV through CCR3- or CCR5-dependent mechanisms (40) also suggests that the clinical spectrum of HIV disease may also depend on the CCR5 genotype.

Thus, complex interactions between virus and host chemokine receptor genotype and virus and cell chemokine receptor phenotype exist. Studies to elucidate the interaction of these genotypes with each other, with virus phenotypes, and with other factors that influence the course of HIV disease are ongoing (24,25). Preliminary data from the Multi Center AIDS Cohort Study suggest that the effect of CCR5 and HLA genotype on survival or disease course are independent and that the effect of HLA on disease outcomes may be greater than the effect of CCR5 genotype (54).

### Population Studies of CCR5 Genotypes

The distribution of the 32bp deletion in different populations is summarized in Table 2. Among Caucasians in North America or Europe, the prevalence of homozygosity for the 32 bp deletion is approximately 1%, and 10% to 20% are heterozygous. In smaller numbers of non-Caucasians, heterozygosity is found in approximately 6% of African-Americans, 7% of Hispanics, 13% of Native Americans, and fewer than 1% of Asians (17,19; CDC, unpub. data). Several studies of non-Caucasian populations including persons from parts of Africa (Zaire, Burkina Faso, Cameroon, Senegal, Benin, Uganda, Rwanda, Kenya, Malawi, Tanzania, Sierre Leone) (12,16,19), Haiti (12), parts of Asia (Thailand, India, China, Korea, Japan, the Philippines) (12,16,19; CDC, unpub. data), and Venezuela (9) have not found the 32 bp deletion among the HIV-infected or -uninfected persons tested (Table 2). One additional study (of more than 3,000 persons) found similar global distributions of CCR5 genotypes (and noted a decreasing frequency from north to south in Europe and Asia).[2]

### Other Chemokine Receptor Polymorphisms

Several other point mutations in CCR5 have been observed (17), but their population prevalence and their role in HIV infection or disease progression have not been reported. No polymorphisms in CXCR4 have been reported to date. CCR3 has two alleles (S or T at position 276) (55-57), and similarly, the population prevalence of these alleles and their role in HIV infection have not yet been determined.

### Implications for Management and Understanding of the Global HIV Epidemic

The discovery of the role of chemokine receptors as coreceptors, along with CD4, for HIV entry has led to a burgeoning of public and private research in HIV pathogenesis, new therapies, and new approaches to vaccines (52,58). For example, in addition to the classification of HIV types on the basis of genetic or neutralization phenotype relatedness, a new virus classification made on the basis of their receptor use has emerged. New therapies based on receptor mimics, receptor ligands, chemokines, or chemokine analogs are being developed and tested in vitro. Genetic engineering with chemokine receptors is being used to develop animal models for HIV transmission and disease progression. And, in the arena of vaccines, a new concept has developed: the induction of high levels of HIV-blocking chemokines such as RANTES, MIP-1α, and MIP-1ß as desirable properties of an HIV vaccine.

New treatments and prevention measures for HIV raise ethical, social, and legal issues that also relate to a number of other infectious and chronic diseases (e.g., malaria, breast cancer, and diabetes) as new genes influencing the risk for these diseases are discovered. Should all

[1]Garred P, Eugen-Olsen J, Iversen AKN, Benfield TL, Svejgaard A, Hofmann, B, the Copenhagen AIDS Study Group. Dual effect of CCR5 Δ32 gene deletion in HIV-1-infected patients. Lancet 1997; 349:1884.

[2]Martinson JJ, Chapman NH, Rees DC, Lui Y-T, Clegg JB. Global distribution of the CCR5 gene 32-basepair deletion [letter]. Nature Genetics 1997;16:100-103.

persons at risk for HIV infection be tested for CCR5 gene polymorphism? Should persons homozygous for the Δ32 CCR5 genotype receive specific counseling regarding their risk for HIV and other sexually transmitted infections? If a person is heterozygous and infected with HIV, should HIV treatment be altered? Could knowledge of one's genotype favorably or unfavorably influence health insurance, life insurance, or employment opportunities? As a result of these questions, educational materials have become available for the public and public health professionals.[3]

Another finding that provoked discussion was the uneven distribution of the genotype across geographic and racial groups. One hypothesis was that some previous epidemic, restricted to Europe, had led to a survival advantage among persons homozygous or heterozygous for the CCR5 Δ32 genotype and had resulted in a concentration of the CCR5 Δ32 genotype in persons of European ancestry. The "black death" of Northern Europe (1347-1350) and other large epidemics have been used as examples (59,60). Thus, certain populations appear to have an increased survival advantage in the new HIV epidemic. Conversely, populations with lower prevalence of the Δ32 CCR5 genotype might be expected to have a higher prevalence of HIV infection or a more rapid course of the epidemic. In the United States, a greater risk for HIV infection has been found among African-Americans than among Caucasians, when known risk factors, including social class, were controlled (61). The prevalence of the Δ32 CCR5 allele is lower in African-Americans than in Caucasians. While increasing HIV prevalence in parts of Asia and Africa may be attributed to social and demographic factors, as well as differences in the phenotype of circulating viruses (62), the racial distribution of HIV risk raises the possibility that differences in the distribution of the Δ32 CCR5 allele or other heritable host factors might influence the rate of transmission or the speed of the epidemic in different racial groups.

### Conclusions

These findings have defined a new role for a gene that normally controls cell migration; have identified new alleles of the gene that determines whether someone becomes infected with HIV and how the disease progresses once infection has occurred; and have identified several other new HIV-receptors that mediate viral entry into different cell types. The findings have precipitated new classifications of HIV phenotypes by cell tropism and receptor use and opened new areas for drug and vaccine development. These findings have also enhanced other research: studies of host factors affecting the acquisition and clinical course of infectious diseases, the differential distribution of genetic characteristics in populations and their potential effects on health status, the translation of genetics research into public health measures, and the social implications of genetic differences affecting illness and death caused by infectious and other diseases.

### Acknowledgments

We thank J.S. McDougal and Renu Lal for critical discussion, Clair Kiernan for assistance with graphics, and Wendy Paris for secretarial help.

Dr. McNicholl is a scientist in the Immunology Branch, Division of AIDS, STD, TB, and Laboratory Research, NCID, CDC, and an Assistant Professor of Medicine (Rheumatology) and Pathology at Emory University. Her research includes immunogenetics and T cell antigen recognition in infectious and autoimmune diseases, and their application to vaccines. In collaboration with other scientists at CDC and in Thailand, she is investigating immunologic and genetic explanations for apparent resistance to HIV of highly exposed persistently seronegative commercial sex workers in Thailand. She also serves as the NCID coordinator for CDC genetics activities.

### References

1. World Health Organization. Acquired immunodeficiency syndrome (AIDS)—November 20, 1996. Wkly Epidemiol Rec 1996; 48:361.
2. Steel CM, Ludlam CA, Beatson D, Peutherer JF, Cuthbert RJG, Simmonds P, et al. HLA haplotype A1 B8 DR3 as a risk factor for HIV-related disease. Lancet 1988;1:1185-8.
3. Kaslow RA, Carrington M, Apple R, Park L, Munoz A, Saah AJ, et al. Influence of combinations of human major histocompatibility complex genes on the course of HIV-1 infection. Nat Med 1996;2:405-11.
4. McNeil AJ, Yap PL, Gore SM, Brettle RP, McCol M, Wyld R, et al. Association of HLA types A1-B8-DR3 and B27 with rapid and slow progression of HIV disease. QJM 1996;89:177-85.
5. Hill AVS. HIV and HLA: confusion or complexity? Nat Med 1996;2:395-400.
6. Malkovsky M. HLA and natural history of HIV infection. Lancet 1996;348:142-3.

[3]Centers for Disease Control and Prevention. Facts about CCR5 and protection against HIV-1 infection; 1997.

7. Rowland-Jones S, Sutton J, Ariyoshi K, Dong T, Gotch F, McAdam S, et al. HIV-specific cytotoxic T-cells in HIV-exposed but uninfected Gambian women. Nature Medicine 1995;1:59-64.
8. Paxton WA, Martin SR, Tse D, O'Brient TR, Skurnick J, VanDevanter NL, et al. Relative resistance to HIV-1 infection of CD4 lymphocytes from persons who remain uninfected despite multiple high-risk sexual exposures. Nat Med 1996;2:412-7.
9. Liu R, Paxton WA, Choe S, Ceradini D, Martin SR Horuk R, et al. Homozygous defect in HIV-1 co-receptor accounts for resistance of some multiply-exposed individuals to HIV-1 infection. Cell 1996;86:367-77.
10. Fowke KR, Nagelkerke NJD, Kimani J, Simonsen JN, Anzala AO, Bwayo JJ, et al. Resistance to HIV-1 infection among persistently seronegative prostitutes in Nairobi, Kenya. Lancet 1996;348:1347-51.
11. Stephens H, Beyrer C, Mastro T, Nelson KE, Klaythong R, Kunachiwa W, et al. HLA class I alleles in a cohort of HIV-1 exposed, persistently seronegative (HEPS) sex workers (CSWs) in Northern Thailand. In: Proceedings of the 3rd Conference on Retroviruses and Opportunistic Infections; 1996 January. Washington (DC): American Society for Microbiology; 1996.
12. Huang Y, Paxton WA, Wolinsky SM, Neumann AU, Zhang L, He T, et al. The role of a mutant CCR5 allele in HIV-1 transmission and disease progression. Nat Med 1996;2:1240-3.
13. Fowke K, Slaney LA, Simonsen JN, Nagelkerke N, Nath A, Anzala AO, et al. HIV-1 resistant prostitutes: an innate mechanism. In: Proceedings of the 1st National Conference on Human Retroviruses; Dec 12–16. Washington (DC): American Society for Microbiology; 1993; p. 82.
14. Plummer FA, Fowke K, Nagelkerke NDJ, Simonsen JN, Bwayo J, Ngugi E, et al. Evidence of resistance to HIV among continuously exposed prostitutes in Nairobi, Kenya. In: Abstracts of the 9th International Conference on AIDS; Berlin 1993 June 6–11; WS-A07-3. Sponsored by the International AIDS Society and World Health Organization.
15. Rowland-Jones SL, McMichael A. Immune responses in HIV-exposed seronegatives: have they repelled the virus? Curr Opin Immunol 1995;7:448-55.
16. Samson M, Libert F, Doranz BJ, Rucker J, Liesnard C, Farber CM, et al. Resistance to HIV-1 infection in Caucasian individuals bearing mutant alleles of the CCR-5 chemokine receptor gene. Nature 1996;382:722-5.
17. Dean M, Carrington M, Winkler C, Huttley GA, Smith MW, Allikmets R, et al. Genetic restriction of HIV-1 infection and progression to AIDS by a deletion of the CKR5 structural gene. Science 1996;273:1856-62.
18. Michael NL, Chang G, Louie LG, Mascola JR, Dondero D, Birx DL, et al. The role of viral phenotype and CCR-5 gene defects in HIV-1 transmission and disease progression. Nat Med 1997;3:338-40.
19. Zimmerman PA, Bucklerwhite A, Alkhatib G, Spalding T, Kubofcik J, Combadiere C. Inherited resistance to HIV-1 conferred by an inactivating mutation in CC chemokine receptor 5—studies in populations with contrasting clinical phenotypes, defined racial background, and quantified risk. Mol Med 1997;3:23-36.
20. Biti R, French R, Young J, Bennetts B, Stewart G. HIV-1 infection in an individual homozygous for the CCR5 deletion allele. Nat Med 1997;3:252-3.
21. O'Brien TR, Winkler C, Dean M, Nelson JAE, Carrington M, Michael NL, et al. HIV-1 infection in a man homozygous for CCR5 Δ32. Lancet 1997;349:1219
22. Theodorou I, Meyer L, Magierowska M, Katlama C, Rouzious C, Seroco Study Group. HIV-1 infection in an individual homozygous for CCR5Δ32. Lancet 1997;349:1219-20.
23. Eugenolsen J, Iverson AKN, Garred P, Koppelhus U, Pedersen C, Benfield TL, et al. Heterozygosity for a deletion in the CKR-5 gene leads to prolonged AIDS-free survival and slower CD4 T-cell decline in a cohort of HIV-seropositive individuals. AIDS 1997;11:305-10.
24. Garred P, Madson HO, Balslev U, Hofmann B, Gerstoft J, Svejgaard A. Susceptibility to HIV infection and progression of AIDS in relation to variant alleles of mannose-binding lectin. Lancet 1997;349:236-40.
25. Brinkman BMN, Keet IPM, Miedema F, Verweij CL, Klein M. Polymorphisms within the human tumor necrosis factor-α promoter region in human immunodeficiency virus type 1-seropositive persons. J Infect Dis 1997;375:188-90.
26. Khoo SH, Pepper L, Snowden N, Hajeer AH, Vallely P, Wilkins EG, et al. Tumor necrosis factor c2 microsatellite allele is associated with the rate of HIV disease progression. AIDS 1997;11:423-8.
27. Murphy PM. Chemokine receptors: structure, function and role in microbial pathogenesis. Cytokine Growth Factor Rev 1996;7:47-64.
28. Napolitano M, Zingoni A, Bernardini G, Spinetti G, Nista A, Storlazzi C, et al. Molecular cloning of TER1, a chemokine receptor-like gene expressed by lymphoid tissues. J Immunol 1996;157:2759-63.
29. Miller LH. Impact of malaria on genetic polymorphism and genetic diseases in Africans and African Americans. Proc Natl Acad Sci U S A 1997;91:2415-9.
30. Tournamille C, Colin Y, Cartron JP, Le Van Kim C. Disruption of a GATA motif in the Duffy gene promoter abolishes erythroid gene expression in Duffy-negative individuals. Nat Genet 1995;10:224-8.
31. Pleskoff O, Treboute C, Brelot A, Heveker N, Seman M, Alizon M. Identification of a chemokine receptor encoded by human cytomegalovirus as a cofactor for HIV-1 entry. Science 1997;276:1874-8.
32. Cocchi F, DeVico AL, Garzine-Demo A, Arya SK, Gallo RC, Lusso P. Identification of RANTES, MIP-1α and MIPß as the major HIV-suppressive factors produced by CD8+ T cells. Science 1995;270:1811-5.
33. Deng HK, Liu R, Ellmeier W, Choe S, Unutmaz D, Burkhart M, et al. Identification of a major co-receptor for primary isolates of HIV-1. Nature 1996;381:661-6.
34. Feng Y, Broder CC, Kennedy PE, Berger EA. HIV-1 entry cofactor—functional CDNA cloning of seven—transmembrane, G protein-coupled receptor. Science 1996;272:872-7.
35. Samson M, Labbe O, Mollereau C, Vassart G, Parmentier M. Molecular cloning and functional expression of a new human CC-chemokine receptor gene. Biochemistry 1996;35:3362-6.

36. Dragic T, Litwin V, Allaway GP, Martin SR, Huang YX, Nagashima KA, et al. HIV-1 entry into CD4(+) cells is mediated by the chemokine receptor CC-CKR-5. Nature 1996;381:667-73.
37. Alkhatib G, Combadiere C, Broder CC, Feng Y, Kennedy PE, Murphy PM, et al. CC CKRS—A RANTES, MIP-1-α, MIP-1ß receptor as a fusion cofactor for macrophage-tropic HIV-1. Science 1996;272:1955-8.
38. Choe H, Farzan M, Sun Y, Sullivan N, Rollins B, Ponath PD, et al. The ß-chemokine receptors CCR3 and CCR5 facilitate infection by primary HIV-1 isolates. Cell 1996;85:1135-48.
39. Doranz BJ, Rucker J, Yi YJ, Smyth RJ, Samson M, Peiper SC, et al. A dual-tropic primary HIV-1 isolate that uses fusin and the ß-chemokine receptors CKR-5, CKR-3 and CKR-2b as fusion cofactors. Cell 1996;85:1149-58.
40. He J, Chen Y, Farzan M, Choe H, Ohagen A, Gartner S, et al. CCR3 and CCR5 are co-receptors for HIV-1 infection of microglia. Nature 1997;385:645-9.
41. Bleul CC, Wu L, Hoxie JA, Springer TA, Mackay CR. The HIV coreceptors CXCR4 and CCR5 are differentially expressed and regulated on human T lymphocytes. Proc Natl Acad Sci U S A 1997;94:1925-30.
42. Simmons G, Wilkinson D, Reeves JD, Dittmar MT, Beddows S, Weber J, et al. Primary, syncytium-inducing human immunodeficiency virus type 1 isolates are dual-tropic and most can use either lestr or CCR5 as coreceptors for virus entry. J Virol 1996;70:8355-60.
43. Zhang L, Huang Y, He T, Cao Y, Ho DD. HIV-1 subtype and second-receptor use. Nature 1996;383:768.
44. Wain-Hobson S. One on one meets two. Nature 1996;384:117-8.
45. Cocchi F, DeVico AL, Garzino-Demo A, Cara A, Gallo RC, Lusso P. The V3 domain of the HIV-1 gp 120 envelope glycoprotein is critical for chemokine-mediated blockade of infection. Nat Med 1996;2:1244-7.
46. Wu L, Gerard NP, Wyatt R, Choe H, Parolin C, Ruffing N, et al. CD4-induced interaction of primary HIV-1 gp120 glycoproteins with the chemokine receptor CCR-5. Nature 1996;384:179-83.
47. Trkola A, Dragic T, Arthos J, Binley JM, Olson WC, Allaway GP, et al. CD4-dependent, antibody-sensitive interactions between HIV-1 and its co-receptor CCR5. Nature 1996;384:184-7.
48. Rucker J, Samson M, Doranz BJ, Libert F, Berson JF, Yi Y, et al. Regions in ß-chemokine receptors CCR5 and CCR2b that determine HIV-1 cofactor specificity. Cell 1996;87:437-46.
49. Atchison RE, Gosling J, Monteclaro FS, Franci C, Digilio L, Charo IF, et al. Multiple extracellular elements of CCR5 and HIV-1: dissociation from response to chemokines. Science 1996;274:1924-6.
50. Lapham C, Ouyang J, Chandrasekhar B, Nguyen N, Dimitrov D, Golding H. Evidence for cell-surface association between fusin and the CD4-gp 120 complex in human cell lines. Science 1996;274:602-5.
51. Oravecz T, Pall M, Norcross MA. ß-Chemokine inhibition of monocytotropic HIV-1 infection. Interference with a postbinding fusion step. J Immunol 1996;157:1329-32.
52. Paxton WA, Dragic T, Koup RA, Moore JP. Perspective—research highlights at the Aaron Diamond AIDS Research Center—the beta-chemokines, HIV type 1 second receptors, and exposed uninfected persons. AIDS Res Hum Retroviruses 1996;12:1203-7.
53. Wu L, Paxton WA, Kassam N, Ruffing N, Rottman JB, Sullivan N, et al. CCR5 levels and expression pattern correlate with infectability by macrophage-tropic HIV-1, in vitro. J Exp Med 1997;185:1681-91.
54. Kaslow RA, Koup R, Zimmerman P, Dean M, Naik E, Enger C, et al. HLA scoring profile (HSP) and CCR5 deletion heterozygosity as predictors of AIDS in seroconverters. In: Proceedings of the 4th Conference on Retroviruses and Opportunistic Infections; Jan 22–26. Washington (DC): American Society of Microbiology: 1997; p. 69.
55. Combadiere C, Ahuja SK, Murphy PM. Cloning and functional expression of a human eosinophil CC chemokine receptor. J Biol Chem 1996;271:11034.
56. Daugherty BL, Siciliano SJ, DeMartino JA, Malkowitz L, Sirotina A, Springer MS. Cloning, expression, and characterization of the human eosinophil eotaxin receptor. J Exp Med 1996;183:2349-54.
57. Ponath PD, Qin S, Post TW, Wang J, Wu L, Gerard NP, et al. Molecular cloning and characterization of a human eotaxin receptor expressed selectively on eosinophils. J Exp Med 1996;183:2437-48.
58. D'Souza MP, Harden VA. Chemokines and HIV-1 second receptors. Nat Med 1996;2:1293-300.
59. New AIDS study reveal startling immunity data. Kolata G. The New York Times. 1996; September 27, 1996. p. A13 New York. The New York Times.
60. Kolata G. Geneticists seek to understand why disease genes spread. The New York Times 1996; Sect. B:5-9.
61. Easterbrook PJ, Chmiel JS, Hoover DR, Saah AJ, Kaslow RA, Kingsley LA, et al. Racial and ethnic differences in human immunodeficiency virus type 1 (HIV-1) seroprevalence among homosexual and bisexual men. The multicenter AIDS cohort study. Am J Epidemiol 1993;138:415-29.
62. Soto-Ramirez LE, Renjifo B, McLane MF, Marlink R, O'Hara C, Sutthent R, et al. HIV-1 Langerhans' cell tropism associated with heterosexual transmission of HIV. Science 1996;271:1291-3.
